# Clinical and Multivariate Predictors of Headaches Attributed to Rhinosinusitis in Pediatric Patients: A Comparative Study with Migraine and Tension-Type Headache

**DOI:** 10.3390/children12111557

**Published:** 2025-11-17

**Authors:** Seung Beom Han, Eu Gene Park, Ji Yoon Han

**Affiliations:** 1 Department of Pediatrics, College of Medicine, The Catholic University of Korea, Seoul 06591, Republic of Korea; beomsid@catholic.ac.kr (S.B.H.); eugene.park@catholic.ac.kr (E.G.P.); 2 Department of Pediatrics, Bucheon St. Mary’s Hospital, College of Medicine, The Catholic University of Korea, Bucheon 14647, Republic of Korea; 3 Department of Pediatrics, Incheon St. Mary’s Hospital, College of Medicine, The Catholic University of Korea, Incheon 21431, Republic of Korea; 4 Department of Pediatrics, Daejeon St. Mary’s Hospital, College of Medicine, The Catholic University of Korea, Daejeon 34943, Republic of Korea

**Keywords:** headache attributed to rhinosinusitis, migraine, tension-type headache

## Abstract

**Highlights:**

**What are the main findings?**

- Pediatric headache attributed to rhinosinusitis (HRS) was often misdiagnosed as migraine or tension-type headache, especially in younger children.
- Multivariate analysis identified distinct predictors of HRS, including nasal and auditory symptoms, allergic rhinitis, and family history of HRS.

**What are the implications of the main finding?**

- Incorporating otolaryngologic and allergic features into diagnostic evaluation can improve accuracy and reduce misclassification.
- These predictors may help clinicians avoid unnecessary neuroimaging and provide timely, targeted therapy for pediatric patients.

**Abstract:**

Background/Objectives: Headache attributed to rhinosinusitis (HRS) is uncommon in children but often misdiagnosed as migraine or tension-type headache (TTH). Overlapping phenotypes, incidental sinus findings on neuroimaging, and limited communication in younger patients complicate diagnosis and lead to inappropriate treatment. Methods: We retrospectively analyzed 3065 pediatric patients (<19 years) presenting with headache at two tertiary neurology clinics (2014–2023) with ≥1 year follow-up. Headaches were classified by ICHD-3 criteria. HRS diagnosis required radiologic sinus pathology and ≥50% improvement within 72 h of antibiotic or decongestant therapy. Demographic, clinical, neuroimaging, and family history data were collected. Symptom profiling used principal component analysis (PCA) and k-means clustering; multivariate logistic regression identified independent predictors. Results: Of 3065 patients, 32.7% had migraines, 15.5% TTH, and 4.5% HRS. Nearly one-third of HRS cases were initially misclassified. Compared with migraine and TTH, HRS patients were younger (median 9 years), more often male, and enriched in preschool age. Independent predictors included shorter duration (<1 h; OR 0.62), higher intensity (OR 2.165), nasal symptoms (OR 9.836), hearing impairment (OR 22.52), allergic rhinitis (OR 8.468), and family history of HRS (OR 32.602) (all *p* < 0.001). PCA showed overlap but distinct clustering: HRS was characterized by sinonasal and otologic features, whereas migraine clustered around sensory hypersensitivity. Conclusions: Pediatric HRS shows distinct predictors—young age, acute severe headache, nasal and auditory symptoms, allergic history, and family history—despite overlap with migraine and TTH. Structured use of these predictors with otolaryngologic assessment may improve diagnostic accuracy, reduce misclassification, and avoid unnecessary neuroimaging or inappropriate therapy.

## 1. Introduction

Headache attributed to rhinosinusitis (HRS) is relatively uncommon in pediatric and adolescent populations, with an estimated diagnostic rate ranging from 3% to 11% among headache sufferers [1,2,3,4]. In comparison, primary headache disorders such as migraine and tension-type headache (TTH) are considerably more prevalent. Meta-analytic data indicate that migraine affects approximately 11% of children and adolescents, while TTH has a pooled prevalence of around 17% [5,6,7,8]. Although the International Classification of Headache Disorders (ICHD-3) provides distinct diagnostic criteria, clinical differentiation of headache subtypes in children often remains challenging due to overlapping symptoms, age-dependent expression of pain, and limited communication ability [9].

Misdiagnosis is particularly common between HRS and primary headache disorders, with studies reporting that up to 50–80% of pediatric patients initially diagnosed with sinus-related headaches were later reclassified as having migraine or TTH after specialist evaluation [10,11]. Furthermore, incidental paranasal sinus abnormalities on neuroimaging are detected in up to 30% of asymptomatic children, contributing to diagnostic uncertainty and frequent inappropriate antibiotic use [12,13].

Migraine in pediatric patients may present with bilateral pain, short duration, and more prominent gastrointestinal symptoms compared to adults, whereas TTH typically manifests with pressure-like pain and fewer autonomic features [2,14]. HRS are frequently associated with facial pain or pressure, nasal symptoms, and positional aggravation, but its phenotypic overlap with migraine and TTH often obscures accurate diagnosis in routine practice. Given these diagnostic ambiguities, reliance solely on conventional clinical criteria may lead to misclassification and suboptimal treatment. Recent studies have advocated for a data-driven approach to headache classification, incorporating symptom profiling, cluster analysis, and machine learning techniques to better define phenotypic subgroups beyond traditional diagnostic boundaries [13,15]. The distinction between headache attributed to rhinosinusitis (HRS) and primary headaches such as migraine and tension-type headache was clarified based on the ICHD-3 criteria. HRS typically presents with dull or pressure-like pain associated with nasal symptoms, whereas migraine is characterized by pulsating pain with sensory hypersensitivity.

Therefore, this study aimed to comprehensively analyze the clinical characteristics of pediatric patients with migraine, TTH, and HRS. In addition, we sought to identify multivariate clinical and symptom-based predictors that can effectively distinguish HRS from primary headache disorders, thereby improving diagnostic precision and facilitating appropriate clinical management

## 2. Materials and Methods

### 2.1. Study Design and Participants

This retrospective cohort study included pediatric patients who visited the pediatric neurology outpatient clinics of Incheon St. Mary’s Hospital and Daejeon St. Mary’s Hospital, affiliated with the Catholic University of Korea, from March 2014 to December 2023. To ensure diagnostic accuracy and clinical relevance, only patients with at least 1 year of follow-up after their initial visit were included. Data extraction and verification were performed independently by two pediatric neurologists using a standardized case report form. Any discrepancies in classification or variable coding were resolved by consensus review to minimize diagnostic bias and ensure reproducibility. Eligible participants were children and adolescents (<19 years) presenting with headache as their primary complaint and undergoing standardized headache assessment including detailed clinical history, neurological examination, and neuroimaging.

Exclusion criteria were (1) known systemic diseases (e.g., autoimmune, metabolic, or oncologic disorders), (2) intracranial space-occupying lesions, (3) significant head trauma within 3 months prior to the initial visit, (4) neurologic disorders unrelated to primary headache, (5) chronic use of medications known to affect headache characteristics (e.g., corticosteroids, valproic acid, or tricyclic antidepressants), (6) secondary headache disorders other than rhinosinusitis, (7) incomplete medical records or <1 year of follow-up, and (8) developmental or cognitive disorders precluding reliable symptom reporting.

### 2.2. Data Collection and Variables

Demographic and clinical data were extracted from electronic medical records, including age, sex, headache onset pattern, duration, pain location, and intensity (assessed using a 0–10 numeric rating scale), frequency, associated symptoms (e.g., gastrointestinal symptoms such as nausea/vomiting; neurological manifestations including visual disturbances and dizziness), headache triggers (e.g., sleep deprivation or specific foods), allergy history (e.g., allergic rhinitis, or asthma), and family history of migraine or other headache disorders. Neuroimaging findings (brain MRI or CT) were reviewed where available. Sinusitis was radiologically confirmed by mucosal thickening ≥ 3 mm or air-fluid levels in the paranasal sinuses, and findings were graded using the Lund-Mackay scoring system when applicable. Cases with missing key data (<5%) were excluded from variable-specific analyses. Headache duration was defined as the typical attack length reported by the patient or caregiver. Pain intensity was measured on a 0–10 scale and classified as mild (1–3), moderate (4–6), or severe (7–10). Other categorical variables such as pain type and triggers were coded as present (1) or absent (0) for analysis.

### 2.3. Headache Classification

Headaches were classified according to ICHD-3. A diagnosis HRS required (1) a temporal relationship between headache onset and documented sinus pathology, defined as radiologic evidence of mucosal thickening ≥ 3 mm or air-fluid levels on paranasal sinus imaging, and (2) ≥50% reduction in headache severity within 72 h of antibiotic or decongestant therapy. Cases without adequate clinical–radiologic correlation or therapeutic response were categorized into the non-HRS group, comprising migraine and TTH based on ICHD-3 criteria. All classifications were independently reviewed by two pediatric neurologists, with discrepancies resolved by consensus.

### 2.4. Statistical and Analytical Methods

Symptom profiles were binarized (present = 1, absent = 0), with missing values (<5%) imputed as absent. Normality of continuous variables was assessed using the Shapiro–Wilk test, and appropriate parametric (Student’s *t*-test) or non-parametric (Mann–Whitney U) tests were applied. Continuous variables were summarized as mean ± SD or median (IQR). Group differences were assessed using appropriate parametric or non-parametric tests (Student’s *t*-test, Mann–Whitney U test, chi-square, or Fisher’s exact test) with Bonferroni correction applied to adjust for multiple comparisons. Principal component analysis (PCA) and k-means clustering were applied to identify data-driven headache subtypes independent of diagnostic labels, and the first two principal components were visualized.

Multivariate logistic regression was used to identify independent predictors of HRS, adjusting for clinically relevant covariates (sex, age at diagnosis, headache localization, duration, frequency, intensity, associated symptoms, and family history). Multicollinearity was assessed using VIF (threshold > 5), and model fit was evaluated using the Hosmer–Lemeshow test. Odds ratios (ORs) with 95% confidence intervals (CIs) and *p*-values were reported. Effect sizes were not calculated because most variables were categorical or non-normally distributed. Model fit was checked by the Hosmer–Lemeshow test, and collinearity by VIF (<5). Analyses were conducted using IBM SPSS Statistics v21.0 (IBM Corp., Armonk, NY, USA) and Python v3.11 (Python Software Foundation, Wilmington, DE, USA), with *p* < 0.05 considered statistically significant.

### 2.5. Ethical Considerations

This study was approved by the Institutional Review Board (IRB) of the Catholic Medical Center (OC23RASI0159 and DC24RASI0047; Seoul, Republic of Korea) and conducted in accordance with the Declaration of Helsinki and ICH-GCP guidelines. The IRB granted a waiver of informed consent due to the retrospective nature of the study and the use of anonymized data to ensure patient confidentiality.

## 3. Results

### 3.1. Study Population

A total of 3065 pediatric patients with headache were included (1409 males and 1656 females). The median age at diagnosis was 11 years (interquartile range [IQR]: 8–14; range: 2–19). Among these, 32.7% were diagnosed with migraine, 15.5% with TTH, and 4.5% with HRS. For subgroup analyses, 1140 migraine patients, 474 TTH patients, and 137 HRS patients were compared (Table 1 and Table 2). Of the 137 patients ultimately diagnosed with HRS, 13 (27.7%) were initially misclassified as having primary headache disorders, including migraine (n = 10, 21.3%) and TTH (n = 4, 6.4%).

### 3.2. Comparison Between HRS and Migraine

The HRS group was significantly younger at diagnosis compared with migraine (median: 9 vs. 12 years, interquartile range [IQR]: 6–11 vs. 9–14, *p* < 0.001) and exhibited a higher proportion of males (58.1% vs. 44.5%, *p* < 0.001). Preschool-aged children (≤6 years) were markedly overrepresented in HRS (28.5% vs. 7.1%), whereas adolescents predominated in migraine (53.4% vs. 14.6%, both *p* < 0.001).

Headache onset patterns differed significantly (*p* < 0.001): acute onset (31.4%) and chronic non-progressive onset (44.5%) were more frequent in HRS, whereas chronic progressive onset was predominant in migraine (40.4%). Headache duration demonstrated distinct separation: short episodes (<1 h) were more common in HRS (41.6%), while migraine predominantly involved attacks lasting 4–72 h (74.8%, *p* < 0.001).

Headache frequency also differed (*p* < 0.001). HRS was characterized by 2–4 (35.8%) or 4–<15 (36.5%) episodes/month, with no daily headaches reported, whereas migraine exhibited higher daily headache prevalence (13.2%).

Accompanying symptoms were significantly more frequent in migraine (87.5% vs. 56.9%, *p* < 0.001), particularly nausea (74.2% vs. 50.4%), photophobia (68.9% vs. 19.7%), and phonophobia (58.1% vs. 0%). Neurologic manifestations were comparable in prevalence (25.1% vs. 21.9%, *p* = 0.476), but differed in distribution: migraine was associated with visual disturbances (19.6%), whereas HRS was more often linked to hearing impairment (7.3%). Triggering factors were reported by 23.3% of migraine and 18.2% of HRS patients (*p* = 0.218). The overall trigger distribution differed significantly (*p* < 0.001): fatigue was more frequent in HRS (11.0% vs. 4.8%), while migraine was more commonly associated with emotional stress and sensory stimuli, although individual differences did not reach statistical significance.

**Figure 1:**
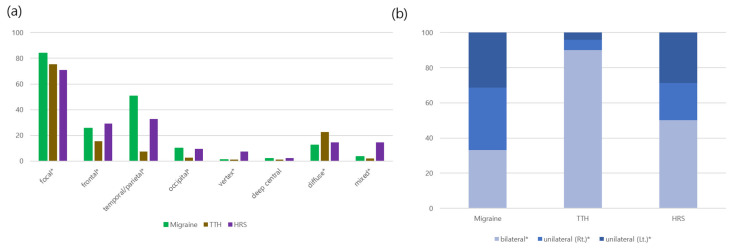
(**a**) Comparison of headache pain locations among patients with migraine, TTH, and HRS. (**b**) Comparison of headache laterality among patients with migraine, TTH, and HRS. Stacked bar graphs show the distribution of bilateral and unilateral (right or left) headache presentations in each group. Asterisks (*) indicate significant differences. Bar graphs display mean ± SD (%) of localization and laterality.

Pain localization (Figure 1) also differed: temporal (62.3% vs. 49.6%) and parietal pain (33.6% vs. 21.9%) were more common in migraine, whereas vertex (15.3% vs. 6.1%) and occipital pain (12.4% vs. 4.9%) were more frequent in HRS (all *p* < 0.05). Laterality further distinguished groups: unilateral pain predominated in migraine (66.8%), whereas HRS displayed a mixed pattern (49.8% bilateral, 42.3% unilateral, *p* < 0.001).

The HRS group showed several distinctive features compared with migraine:

- Younger age at diagnosis (median 9 vs. 12 years, *p* < 0.001).
- Higher proportion of males (58.1% vs. 44.5%, *p* < 0.001).
- More preschool-age patients (28.5% vs. 7.1%, *p* < 0.001).
- Shorter headache duration (<1 h: 41.6% vs. 12.8%, *p* < 0.001).
- Fewer sensory symptoms such as photophobia and phonophobia, but more nasal and auditory symptoms (*p* < 0.001).

### 3.3. Comparison Between HRS and TTH

HRS patients were significantly younger than those with TTH (median: 9 vs. 11 years, interquartile range [IQR]: 6–11 vs. 8–14, *p* < 0.001) and more frequently male (58.1% vs. 48.9%, *p* = 0.035). Preschool-aged children (≤6 years) were markedly overrepresented in HRS (28.5% vs. 13.1%), whereas adolescents predominated in TTH (38.0% vs. 14.6%, both *p* < 0.001).

Headache onset patterns differed significantly (*p* < 0.001). Acute onset was more common in HRS (31.4% vs. 13.7%), while acute recurrent onset characterized TTH (39.9% vs. 13.9%). Chronic non-progressive onset was observed more often in HRS (36.5% vs. 31.2%), while chronic progressive onset frequencies were comparable (18.2% vs. 15.2%). Headache intensity and pain characteristics showed marked differences. Severe pain was more frequent in HRS (26.3% vs. 8.0%, *p* < 0.001), while mild pain predominated in TTH (36.9% vs. 13.1%, *p* < 0.001). Pressure-type pain (72.8% vs. 24.1%, *p* < 0.001) and constant/dull pain (17.5% vs. 7.3%, *p* = 0.005) were typical of TTH, whereas throbbing (24.8% vs. 4.4%, *p* < 0.001) and mixed pain (28.5% vs. 1.5%, *p* < 0.001) were enriched in HRS.

Accompanying symptoms were more prevalent in HRS (56.9% vs. 35.4%, *p* < 0.001), including nausea/vomiting (50.4% vs. 4.6%), abdominal pain (11.0% vs. 3.2%), dizziness (32.1% vs. 20.3%), and nasal symptoms (38.7% vs. 2.3%). Neurologic manifestations were significantly higher in HRS (21.9% vs. 7.8%, *p* < 0.001), with visual disturbances (19.0% vs. 4.4%) and hearing impairment (7.3% vs. 0.2%) particularly notable. Triggering factors were less frequent overall but differed in pattern (*p* < 0.001): fatigue was more common in HRS (11.0% vs. 5.1%), whereas emotional stress predominated in TTH (16.6% vs. 11.0%).

Family history also revealed distinct associations: allergic rhinitis (14.6% vs. 1.3%) and HRS (26.3% vs. 1.1%) were enriched in HRS, whereas familial TTH was more frequent in TTH (24.1% vs. 2.2%, all *p* < 0.001). Notably, migraine family history rates were low and comparable (6.8% vs. 6.6%).

The key differences between HRS and TTH can be summarized as follows:

- Younger age at diagnosis in HRS (median 9 vs. 11 years, *p* < 0.001).
- Higher proportion of males (58.1% vs. 48.9%, *p* < 0.001).
- More preschool-age patients and fewer adolescents (*p* < 0.001).
- Greater headache intensity and more nasal/auditory symptoms (*p* < 0.001).
- Less frequent fatigue as a trigger (*p* = 0.045).

### 3.4. Symptom Profiling and Cluster Analysis

**Figure 2:**
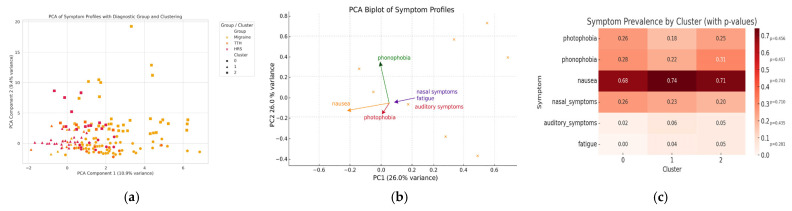
Multivariate symptom profiling across headache subtypes. PCA revealed partial but distinct separation among migraine, TTH, and HRS (**a**). The biplot identified sensory hypersensitivity versus sinonasal–otologic features as the main differentiating axes (**b**). × = individual patient scores; arrows = symptom loadings. Cluster-based heatmap confirmed that nasal and auditory symptoms were predominant in the HRS-dominant cluster (**c**).

Principal component analysis (PCA) of 12 headache-related symptoms demonstrated that the first two components (PC1 and PC2) accounted for 10.9% and 9.4% of the total variance, respectively (Figure 2). In the PCA plot, PC1 mainly reflected sensory hypersensitivity symptoms such as photophobia, phonophobia, and nausea, while PC2 represented sinonasal and otologic features including nasal congestion and hearing impairment. Migraine cases tended to cluster along PC1, HRS along PC2, and TTH showed wide overlap between both axes. These distributions indicate partial separation but substantial overlap among headache subtypes. Migraine cases partially aggregated along PC1, largely driven by sensory hypersensitivity features such as photophobia, phonophobia, and nausea, whereas HRS aligned predominantly along PC2 due to sinonasal symptoms, hearing impairment, and fatigue. TTH exhibited diffuse distribution across both axes, reflecting its overlap with non-specific symptoms. Despite partial aggregation, substantial intergroup overlap was evident, underscoring phenotypic heterogeneity across diagnoses. K-means clustering identified three symptom-based clusters: (1) migraine-dominant with sensory hypersensitivity (photophobia, phonophobia, nausea), (2) HRS-dominant with sinonasal and otologic features (nasal congestion, rhinorrhea, hearing impairment), and (3) mixed profiles characterized by dizziness and fatigue irrespective of diagnosis. Notably, each cluster contained cases from multiple diagnostic categories, highlighting that symptom combinations transcend conventional diagnostic boundaries.

### 3.5. Supplementary Table S1: Three-Group Comparison

Supplementary Table S1 compares migraine, TTH, and HRS across demographic and clinical variables. Age distribution differed significantly (*p* < 0.001): HRS was enriched in preschool-aged children (28.5%), TTH in school-aged children (48.9%), and migraine in adolescents (53.4%). Headache onset patterns varied (*p* < 0.001), with acute and chronic non-progressive onset predominating in HRS, chronic progressive onset in migraine, and acute recurrent onset in TTH. Headache localization differed markedly (*p* < 0.001): focal pain was most frequent in migraine (83.9%), while diffuse pain was more common in TTH (22.6%) and mixed pain in HRS (14.6%). Laterality also showed clear separation (*p* < 0.001): unilateral pain predominated in migraine (66.8%), bilateral pain in TTH (90.0%), and HRS exhibited an intermediate distribution (50.2% bilateral, 42.3% unilateral).

Accompanying symptoms were significantly more prevalent in migraine and HRS compared to TTH, including nausea/vomiting (74.2%, 50.4% vs. 4.6%) and dizziness (34.6%, 32.1% vs. 20.3%). Nasal symptoms were unique to HRS (38.7%, *p* < 0.001). Neurologic manifestations followed a gradient: migraine (25.1%) > HRS (21.9%) > TTH (7.8%). Triggering factors differed significantly (*p* < 0.001): fatigue was most associated with HRS (11.0%), while emotional stress predominated in TTH (16.6%). Family history patterns showed diagnosis-specific clustering: migraine was strongly linked to familial migraine (32.9%), TTH to familial TTH (24.1%), and HRS to allergic rhinitis (14.6%) and familial HRS (26.3%). These findings reinforce distinct demographic and phenotypic profiles while highlighting overlapping features that complicate diagnosis.

### 3.6. Multivariate Predictors of HRS

Multivariate logistic regression (Table 3) identified several independent predictors of HRS. Younger age (OR = 0.71, 95% CI: 0.522–0.967, *p* = 0.030) and male sex (OR = 0.567, 95% CI: 0.334–0.963, *p* = 0.036) were inversely associated with HRS, indicating that younger boys were more likely to be diagnosed with HRS. Shorter headache duration (OR = 0.62, 95% CI: 0.484–0.793, *p* < 0.001) and higher pain intensity (OR = 2.165, 95% CI: 1.380–3.397, *p* = 0.001) were also significant predictors. Otolaryngologic and allergic features showed the strongest associations: nasal symptoms (OR = 9.836, 95% CI: 4.548–21.273), hearing impairment (OR = 22.52, 95% CI: 7.153–70.989), family history of HRS (OR = 32.602, 95% CI: 14.312–74.265), and allergic rhinitis (OR = 8.468, 95% CI: 3.484–20.582) were all highly predictive (all *p* < 0.001). Fatigue also emerged as an independent predictor (OR = 3.935, 95% CI: 1.715–9.029, *p* = 0.001).

In contrast, headache frequency (OR = 1.217, *p* = 0.065), pain location (OR = 1.57, *p* = 0.194), nausea/vomiting (OR = 1.046, *p* = 0.870), photophobia (OR = 0.412, *p* = 0.097), and phonophobia (OR = 0.398, *p* = 0.998) were not independently associated with HRS in the multivariate model.

## 4. Discussion

This study comprehensively compared migraine, TTH, and HRS in a large pediatric cohort, highlighting both shared and distinguishing features. We also identified independent predictive factors for HRS using multivariate analysis, offering clinically relevant tools to improve diagnostic accuracy and inform tailored management strategies in children and adolescents.

### 4.1. Comparison with Previous Studies

Our findings reaffirm prior reports that HRS is more common in younger children and characterized by acute onset, shorter headache duration, and otolaryngologic symptoms such as nasal congestion and rhinorrhea [11,13,16]. In contrast, migraine predominated in adolescents and was associated with longer attack duration, higher frequency, and migrainous features—including throbbing pain, photophobia, phonophobia, and nausea—consistent with its established diagnostic criteria [9]. TTH displayed the expected phenotype of bilateral, pressing-type pain with fewer associated symptoms, aligning with its musculoskeletal pathophysiology [17,18]. The key distinguishing clinical features among migraine, tension-type headache, and headache attributed to rhinosinusitis are summarized in Supplementary Table S2.

However, our study diverges from earlier literature in several respects. Prior studies have linked migraine occurrence to seasonal peaks in winter or spring [19,20] and HRS to allergy-associated seasons [12,21], In contrast, we observed no significant seasonal variation across diagnostic groups, with peak case numbers occurring during summer months. This discrepancy may reflect regional climatic differences, relatively stable allergen exposure patterns, or sociobehavioral factors such as school calendar-related stress or healthcare utilization unique to our population [22]. historical clinical markers such as early-morning headaches or sleep-disrupting pain, which have been proposed to distinguish secondary from primary headaches [11,23], were not discriminative in our cohort. This finding suggests that such features may be less reliable in modern practice, where early neuroimaging, routine otolaryngologic assessment, and broader access to pediatric headache specialists reduce reliance on indirect or historical diagnostic cues.

### 4.2. HRS Versus Primary Headaches: Shared and Distinguishing Features

Notably, 27.7% of HRS patients in our cohort were initially misclassified as primary headache (migraine or TTH), underscoring the substantial clinical overlap and diagnostic challenge, particularly in younger children with limited ability to describe sensory features. This finding is consistent with prior studies reporting that 42–80% of patients initially labeled as ‘sinus headache’ were ultimately diagnosed with migraine following specialist evaluation [17,24]. These data highlight the importance of incorporating sinonasal findings—such as nasal symptoms or hearing impairment—into diagnostic pathways to improve diagnostic accuracy.

Although HRS exhibited distinct features, it also shared several characteristics with migraine and TTH. Neurologic manifestations were unexpectedly frequent in HRS (21.9%), second only to migraine (25.1%), and higher than previously reported [25]. While visual disturbances predominated in migraines, HRS was more often associated with hearing impairment and dizziness. This may be attributable to sinus inflammation and its anatomical proximity to cranial nerves, referred pain mechanisms, or secondary venous congestion [26,27]. Moreover, overlapping autonomic features—such as nasal congestion and rhinorrhea—may arise from shared trigeminovascular activation, further obscuring the boundary between HRS and migraine and contributing to frequent misdiagnosis in both pediatric and adult populations [24,28]. This overlap is especially problematic in young children who cannot reliably articulate sensory or autonomic symptoms, emphasizing the diagnostic value of caregiver reports and comprehensive otolaryngologic assessment.

Pain topography further revealed differentiating clues: temporal and parietal pain were common to both migraine and HRS, whereas vertex and occipital pain were significantly enriched in HRS, consistent with posterior paranasal sinus involvement [29,30,31]. Headache laterality also varied: unilateral pain predominated in migraine, bilateral pain in TTH, while HRS displayed an intermediate mixed pattern [4,32]. These anatomical distinctions may assist differentiation, especially in diagnostically ambiguous cases. Emerging evidence suggests that sinus inflammation in HRS can activate trigeminal nociceptive pathways and perivascular meningeal afferents, resulting in pain patterns that closely mimic migraine. Pro-inflammatory cytokines such as interleukin-6 and tumor necrosis factor-alpha, detected in acute bacterial sinusitis, may sensitize trigeminovascular circuits [33]. Additionally, venous congestion in the cavernous sinus and perisinusoidal regions may produce referred orbital and frontal pain frequently mistaken for migraine [34]. This neuroinflammatory overlap underscores the neurovascular interface linking HRS and migraine and reinforces the need for timely otolaryngology referral and targeted evaluation in diagnostically ambiguous cases.

### 4.3. Clinical Implications and Application of Predictors

While radiologic sinus changes support HRS diagnosis, their non-specificity limits their standalone utility, as incidental sinus opacification is observed in up to 30% of asymptomatic children undergoing brain MRI [35]. Incorporating nasal endoscopy, when feasible, may enhance diagnostic confidence by allowing direct visualization of purulent drainage or mucosal edema [36]. Additionally, validated pediatric sinonasal symptom scales, such as the SN-5 (Sinus and Nasal Quality of Life Survey), can complement headache evaluation by quantifying sinonasal symptom burden and improving diagnostic accuracy [37].

Incorporating the clinical predictors identified in our multivariate analysis—namely, younger age, male sex, shorter headache duration, high pain intensity, nasal symptoms, hearing impairment, fatigue, family history of HRS, and allergic rhinitis—into routine assessment may significantly improve diagnostic precision. These findings align with existing literature linking allergic sensitization and otolaryngologic pathology to HRS [13,21]. For example, a preschool-aged child presenting with acute, severe headache, nasal congestion, and a positive family history of allergic rhinitis should prompt early otolaryngologic referral and consideration of HRS, even in the absence of typical migraine features.

Clinically, these predictors provide a pragmatic framework for triaging pediatric headache patients, reducing reliance on neuroimaging, and avoiding delays in appropriate treatment. Comprehensive nasal and otolaryngologic examination, coupled with systematic inquiry into allergic and familial history, can facilitate early identification of HRS and enable targeted interventions such as antibiotics, decongestants, or allergy management rather than empiric migraine therapy. Based on these independent predictors, a simplified diagnostic algorithm for pediatric headache attributed to rhinosinusitis was developed to assist clinical decision-making (Supplementary Figure S1)

Integrating these predictors into structured triage algorithms may also optimize healthcare resource utilization. Early recognition of HRS can decrease unnecessary neuroimaging, reduce inappropriate migraine pharmacotherapy, and expedite appropriate antibiotic or decongestant initiation. Prior cost-effectiveness analyses support that otolaryngology referral pathways incorporating symptom-based screening can reduce diagnostic delays by 30–40% while lowering imaging costs [38].

Given these diagnostic challenges, clinicians should adopt structured approaches that integrate headache phenotype, otolaryngologic features, allergic/familial history, and environmental context. While prior studies have emphasized triggers such as weather or barometric pressure changes in migraine [39], our cohort demonstrated low rates of weather-related triggers but a strong association with fatigue, highlighting the need for population-specific trigger profiling [40]. Standardized screening tools, including pediatric migraine trigger questionnaires, may further enhance history-taking and contextualize headache triggers across subtypes.

In younger children, limited ability to verbalize headache quality or associated symptoms necessitates greater reliance on caregiver observations, otolaryngologic examination, and indirect behavioral cues. Signs such as irritability, disrupted sleep, or facial tenderness on palpation may serve as surrogate markers of sinus-related pain in non-verbal or preschool-aged patients. Our finding that 28.5% of HRS cases occurred in preschool-aged children underscores the need for age-tailored assessment frameworks and early otolaryngologic involvement in this group.

### 4.4. Integration with Existing Literature

Our findings are consistent with reports highlighting the phenotypic heterogeneity of pediatric headache. Previous studies have reported that 40–60% of pediatric migraine cases are initially misdiagnosed as sinusitis, leading to delayed or inappropriate treatment [26]. PCA and clustering analyses revealed significant overlap between diagnoses, explaining only modest variance (10.9% and 9.4%), supporting the concept of a clinical continuum rather than discrete entities [41,42]. This aligns with emerging machine learning studies advocating for multidimensional classification systems that transcend traditional ICHD criteria. Such models may better capture intermediate phenotypes such as HRS-migraine overlap, facilitating tailored management strategies.

Furthermore, the relatively high rate of neurologic symptoms in HRS suggests that clinicians should not dismiss transient sensory or visual disturbances in the context of suspected sinus-related headache. Rather, these should prompt careful evaluation for otolaryngologic pathology in conjunction with neurologic work-up, avoiding premature assignment to primary headache categories [12,39].

### 4.5. Limitations and Future Directions

Integrating multivariate predictors into predictive nomograms or electronic clinical decision-support tools has the potential to standardize diagnosis across both primary and specialty care. Machine learning approaches—such as random forest and gradient boosting models that incorporate symptom clusters, allergy history, and demographic variables—have shown promise in pediatric headache classification and could be leveraged to further refine HRS detection [43].

This study has several limitations. First, its retrospective design introduces inherent documentation bias, particularly for subjective features such as photophobia, headache timing, and pain descriptors, which may be underreported or inconsistently recorded. Second, otolaryngologic findings were derived primarily from radiology reports rather than validated sinus scoring systems, limiting the granularity of sinonasal assessment. Third, our clustering and PCA analyses were exploratory in nature and accounted for only modest variance, underscoring the need for validation in larger, prospective cohorts. Finally, because our data were derived from a tertiary-care setting, generalizability to primary care or community populations may be limited. Information on medication use was unavailable in our retrospective dataset; therefore, potential similarities in drug response between HRS and migraine could not be evaluated and were noted as a limitation. It should also be noted that sinus-related and primary headaches may coexist in the same patient, and their clinical features can overlap; thus, complete differentiation is not always possible in clinical practice.

Another potential concern is the selection of patients, as most participants were recruited from tertiary pediatric neurology clinics. In the United States and other Western healthcare systems, children with acute headache symptoms are often first evaluated in primary care or emergency settings, and referral to pediatric neurology may be delayed. However, in Korea, patients and caregivers can directly access tertiary centers without requiring primary care referral, and acute headache presentations are commonly seen in pediatric neurology clinics. Thus, our cohort reflects the actual referral patterns in the Korean healthcare system, which should be considered when interpreting the generalizability of our findings.

Future studies should be prospective and multicentric, incorporating standardized allergy testing, validated otolaryngologic scoring systems (e.g., Lund-Mackay or SN-5), and longitudinal headache diaries to improve phenotypic resolution. Additionally, integration of advanced analytical methods, including machine learning algorithms and multi-omics approaches (e.g., transcriptomic or cytokine profiling), could facilitate a mechanistic understanding of the overlap between HRS and primary headaches and enable the development of personalized, phenotype-driven diagnostic frameworks.

## 5. Conclusions

This study identified key clinical predictors that distinguish pediatric headache attributed to rhinosinusitis (HRS) from migraine and tension-type headache. Younger age, male sex, short headache duration, high pain intensity, nasal and auditory symptoms, allergic rhinitis, and family history of HRS were the most reliable indicators. Integrating these predictors into clinical evaluation may reduce misdiagnosis, limit unnecessary neuroimaging, and guide timely otolaryngologic referral. Future multicenter prospective studies using standardized sinonasal scoring and machine-learning–based models are warranted to refine diagnostic algorithms for pediatric HRS.

## Figures and Tables

**Table 1:** Comparison of headache characteristics between the migraine and HRS.

Clinical Characteristics	Migraine (n = 1140)	HRS (n = 137)	*p*-Value
Male (n, %) Female (n, %)	471 (41.3) 669 (58.7)	118 (58.1%) 85 (41.9%)	<0.001
Age (years), median (range)	12 (2–18)	9 (3–17)	<0.001
Age at diagnosis (n, %)			<0.001
Pre-school age (≤6 years)	81 (7.1)	39 (28.5)	<0.001
Children (7–12 years)	450 (39.5)	78 (56.9)	<0.001
Adolescent (13–18 years)	609 (53.4)	20 (14.6)	<0.001
Onset type (n, %)			<0.001
Acute (≤3 months)	221 (19.4)	43 (31.4)	0.002
Acute recurrent (≤3 months)	227 (19.9)	19 (13.9)	0.114
Chronic non-progressive (>3 months)	214 (18.8)	50 (36.5)	<0.001
Chronic progressive (>3 months)	478 (41.9)	25 (18.2)	<0.001
Localization (n, %)			<0.001
Diffuse	144 (12.6)	20 (14.6)	0.607
Localized	956 (83.9)	97 (70.8)	<0.001
Mixed	40 (3.5)	20 (14.6)	<0.001
Duration (n, %)			<0.001
<30 min	146 (12.8)	38 (27.7)	<0.001
30–<60 min	141 (12.4)	35 (25.6)	<0.001
≥1 h	853 (74.8)	64 (46.7)	<0.001
Frequency (n, %)			<0.001
<2/month	131 (11.5)	0	<0.001
2–<4/month	248 (21.8)	49 (35.8)	<0.001
4–<15/month	298 (26.2)	50 (36.5)	0.013
≥15/month	161 (14.1)	38 (27.7)	<0.001
Daily	151 (13.2)	0	<0.001
Intensity (n, %)			0.039
Mild	80 (7.0)	18 (13.1)	0.018
Moderate	749 (65.7)	83 (60.6)	0.274
Severe	311 (27.3)	36 (26.3)	0.883
Sleep disturbance due to headache (n, %)	158 (13.9)	22 (16.1)	0.569
Morning headache (n, %)	368 (32.3)	33 (24.1)	0.064
Characteristics (n, %)			<0.001
Throbbing	727 (63.8)	34 (24.8)	<0.001
Sharp	80 (7.0)	9 (6.6)	0.986
Cramping	24 (2.1)	1 (0.7)	0.440
Prickling	105 (9.2)	10 (7.3)	0.562
Constant/dull	33 (2.9)	10 (7.3)	0.014
Pressure	192 (16.8)	33 (24.1)	0.047
Mixed	34 (3.0)	39 (28.5)	<0.001
Others	49 (4.3)	1 (0.7)	0.072
Accompanied symptoms (n, %)	998 (87.5)	78 (56.9)	<0.001
Nausea/vomiting	796 (69.8)	69 (50.4)	<0.001
Abdominal pain	66 (5.8)	15 (11.0)	0.031
Photophobia	266 (23.3)	12 (8.8)	<0.001
Phonophobia	279 (24.5)	0	<0.001
Dizziness	394 (34.6)	44 (32.1)	0.653
Nasal symptoms *	21 (1.8)	53 (38.7)	<0.001
Neurologic manifestations (n, %)	286 (25.1)	30 (21.9)	<0.001
Gait disturbance	1 (0.09)	2 (1.5)	0.028
Focal weakness	33 (2.9)	3 (2.2)	0.843
Visual disturbance	224 (19.6)	26 (19)	0.942
Auditory symptoms ^#^	10 (0.9)	10 (7.3)	<0.001
Dysarthria/aphasia	9 (0.8)	1 (0.7)	1.000
Dysesthesia	38 (3.3)	2 (2.5)	0.352
Decreased consciousness	10 (0.9)	2 (2.5)	0.842
Seizure	3 (0.3)	0	1.000
Movement symptom ^†^	17 (1.5)	4 (2.9)	0.375
Triggering factors (n, %)	266 (23.3)	25 (18.2)	<0.001
Emotional stress	182 (16.0)	15 (11.0)	0.158
Hunger	7 (0.6)	0	0.759
Weather	37 (3.2)	2 (2.5)	0.376
Fatigue	43 (3.8)	15 (11.0)	<0.001
Exercise	17 (1.5)	4 (2.9)	0.375
Light	11 (1.0)	2 (1.5)	0.924
Noise	8 (0.7)	0	0.681
Smell	16 (1.4)	0	0.323
Season at diagnosis (n, %)			0.694
Spring	288 (25.3)	35 (25.6)	1.000
Summer	365 (32.0)	45 (32.8)	0.921
Fall	296 (26.0)	30 (21.9)	0.353
Winter	191 (16.7)	27 (19.7)	0.454
Family history of migraine (n, %)	368 (32.3)	9 (6.6)	<0.001
Family history of TTH (n, %)	45 (3.9)	3 (2.2)	0.008
Family history of HRS (n, %)	12 (1.0)	36 (26.3)	<0.001
Family history of allergic rhinitis (n, %)	23 (2.0)	20 (14.6)	<0.001

^†^ Tremor, myoclonus; * Rhinorrhea, nasal stuffiness, postnasal drip, snoring; ^#^ Ear fullness, tinnitus. TTH: tension-type headache, HRS: headache attributed to acute rhinosinusitis. *p*-values were derived using chi-square or Fisher’s exact tests for categorical variables and Mann–Whitney U or *t*-tests for continuous variables, as appropriate.

**Table 2:** Comparison of headache characteristics between the tension-type headache and HRS.

Clinical Characteristics	TTH (n = 474)	HRS (n = 137)	*p*-Value
Male, (n, %) Female, (n, %)	232 (48.9) 242 (51.1)	118 (58.1%) 85 (41.9%)	0.035
Age (years), median (range)	11 (3–18)	9 (3–17)	<0.001
Age at diagnosis (n, %)			<0.001
Pre-school age (≤6 years)	62 (13.1)	39 (28.5)	<0.001
Children (7–12 years)	232 (48.9)	78 (56.9)	0.121
Adolescent (13–18 years)	180 (38.0)	20 (14.6)	<0.001
Onset type (n, %)			<0.001
Acute (≤3 months)	65 (13.7)	43 (31.4)	<0.001
Acute recurrent (≤3 months)	189 (39.9)	19 (13.9)	<0.001
Chronic non-progressive (>3 months)	148 (31.2)	50 (36.5)	0.290
Chronic progressive (>3 months)	72 (15.2)	25 (18.2)	0.465
Localization (n, %)			<0.001
Diffuse	107 (22.6)	20 (14.6)	0.056
Localized	357 (75.3)	97 (70.8)	0.340
Mixed	10 (2.1)	20 (14.6)	<0.001
Duration (n, %)			0.289
<30 min	132 (27.9)	38 (27.7)	1.000
30–<60 min	93 (19.6)	35 (25.6)	0.167
≥1 h	249 (52.5)	64 (46.7)	0.270
Frequency (n, %)			<0.001
<2/month	60 (12.7)	0	<0.001
2–<4/month	96 (20.3)	49 (35.8)	<0.001
4–<15/month	146 (30.8)	50 (36.5)	0.249
≥15/month	67 (14.1)	38 (27.7)	<0.001
Daily	105 (22.1)	0	<0.001
Intensity (n, %)			<0.001
Mild	175 (36.9)	18 (13.1)	<0.001
Moderate	261 (55.1)	83 (60.6)	0.294
Severe	38 (8.0)	36 (26.3)	<0.001
Sleep disturbance due to headache (n, %)	53 (11.2)	22 (16.1)	0.166
Morning headache (n, %)	113 (23.8)	33 (24.1)	1.000
Characteristics (n, %)			<0.001
Throbbing	21 (4.4)	34 (24.8)	<0.001
Sharp	1 (0.2)	9 (6.6)	<0.001
Cramping	3 (0.6)	1 (0.7)	1.000
Prickling	27 (5.7)	10 (7.3)	0.624
Constant/dull	83 (17.5)	10 (7.3)	0.005
Pressure	345 (72.8)	33 (24.1)	<0.001
Mixed	7 (1.5)	39 (28.5)	<0.001
Others	8 (1.7)	1 (0.7)	0.677
Accompanied symptoms (n, %)	168 (35.4)	78 (56.9)	<0.001
Nausea/vomiting	22 (4.6)	69 (50.4)	<0.001
Abdominal pain	15 (3.2)	15 (11.0)	<0.001
Photophobia	29 (6.1)	12 (8.8)	0.371
Phonophobia	43 (9.1)	0	<0.001
Dizziness	96 (20.3)	44 (32.1)	0.005
Nasal symptoms *	11 (2.3)	53 (38.7)	<0.001
Neurologic manifestations (n, %)	37 (7.8)	30 (21.9)	<0.001
Gait disturbance	0	2 (1.5)	0.074
Focal weakness	4 (0.8)	3 (2.2)	0.396
Visual disturbance	21 (4.4)	26 (19)	<0.001
Auditory symptoms ^#^	1 (0.2)	10 (7.3)	<0.001
Dysarthria/aphasia	0	1 (0.7)	0.508
Dysesthesia	2 (0.4)	2 (1.5)	0.468
Decreased consciousness	5 (1.1)	2 (1.5)	1.000
Seizure	1 (0.2)	0	1.000
Movement symptom ^†^	3 (0.6)	4 (2.9)	0.078
Triggering factors (n, %)	109 (23)	25 (18.2)	<0.001
Emotional stress	79 (16.6)	15 (11.0)	0.134
Hunger	0	0	-
Weather	15 (3.2)	2 (2.5)	0.439
Fatigue	24 (5.1)	15 (11.0)	0.022
Exercise	8 (1.7)	4 (2.9)	0.572
Light	0	2 (1.5)	0.074
Noise	2 (0.4)	0	1.000
Smell	8 (1.7)	0	0.270
Season at diagnosis (n, %)			0.458
Spring	107 (22.6)	35 (25.6)	0.541
Summer	133 (28.0)	45 (32.8)	0.327
Fall	125 (26.4)	30 (21.9)	0.343
Winger	109 (23.0)	27 (19.7)	0.485
Family history of migraine (n, %)	30 (6.8)	9 (6.6)	1.000
Family history of TTH (n, %)	114 (24.1)	3 (2.2)	<0.001
Family history of HRS (n, %)	5 (1.1)	36 (26.3)	<0.001
Family history of allergic rhinitis (n, %)	6 (1.3)	20 (14.6)	<0.001

^†^ Tremor, myoclonus; * Rhinorrhea, nasal stuffiness, postnasal drip, snoring; ^#^ Ear fullness, tinnitus. TTH: tension-type headache, HRS: headache attributed to acute rhinosinusitis. *p*-values were derived using chi-square or Fisher’s exact tests for categorical variables and Mann–Whitney U or *t*-tests for continuous variables, as appropriate.

**Table 3:** Multivariate analysis of predictive factors for HRS.

Factors	OR	95% Cl	*p*-Value
Male sex	0.567	0.334–0.963	0.036
Age	0.71	0.522–0.967	0.030
Location	1.57	0.794–3.102	0.194
Duration	0.62	0.484–0.793	<0.001
Frequency	1.217	0.988–1.499	0.065
Intensity	2.165	1.380–3.397	0.001
Nausea/vomiting	1.046	0.609–1.796	0.870
Photophobia	0.412	0.145–1.172	0.097
Phonophobia	0.398	0.123–1.165	0.998
Nasal symptoms	9.836	4.548–21.273	<0.001
Auditory symptoms	22.52	7.153–70.989	<0.001
Fatigue	3.935	1.715–9.029	0.001
Family history of HRS	32.602	14.312–74.265	<0.001
Family history of allergic rhinitis	8.468	3.484–20.582	<0.001

HRS: headache attributed to acute rhinosinusitis, OR: odds ratio, Cl: confidence interval. The chi-square test or Fisher’s exact test (for small cell counts) was used for categorical comparisons.

## Data Availability

The data that support the findings of this study are not publicly available due to ethical and privacy restrictions involving pediatric patient records. However, de-identified data may be available from the corresponding author upon reasonable request and with appropriate IRB approval.

## Supplementary Materials

The following supporting information can be downloaded at: https://www.mdpi.com/article/10.3390/children12111557/s1, Table S1: Comparison of headache characteristics between the migraine, TTH and HRS. Table S2: Clinical differences between migraine, TTH, and HRS. Figure S1: Clinical diagnostic algorithm for pediatric HRS.

## Abbreviations

The following abbreviations are used in this manuscript:

HRS	Headache attributed to rhinosinusitis
TTH	Tension-type headache
OR	Odds ratio
CL	Confidence interval
PC	Principal component
ROC	Receiver operating characteristics
ICHD	International classification of headache disorders
IRB	Institutional review board
