# Supplementary material for: Clinical and Multivariate Predictors of Headaches Attributed to Rhinosinusitis in Pediatric Patients: A Comparative Study with Migraine and Tension-Type Headache
Source: Children (Basel). 2025 Nov 17;12(11):1557. doi: 10.3390/children12111557 (PMC12651926; doi:10.3390/children12111557)
Supplement: Supplementary file 1 [file children-12-01557-s001.zip › Suppelement Figure S1_children.pptx]

## Slide 1
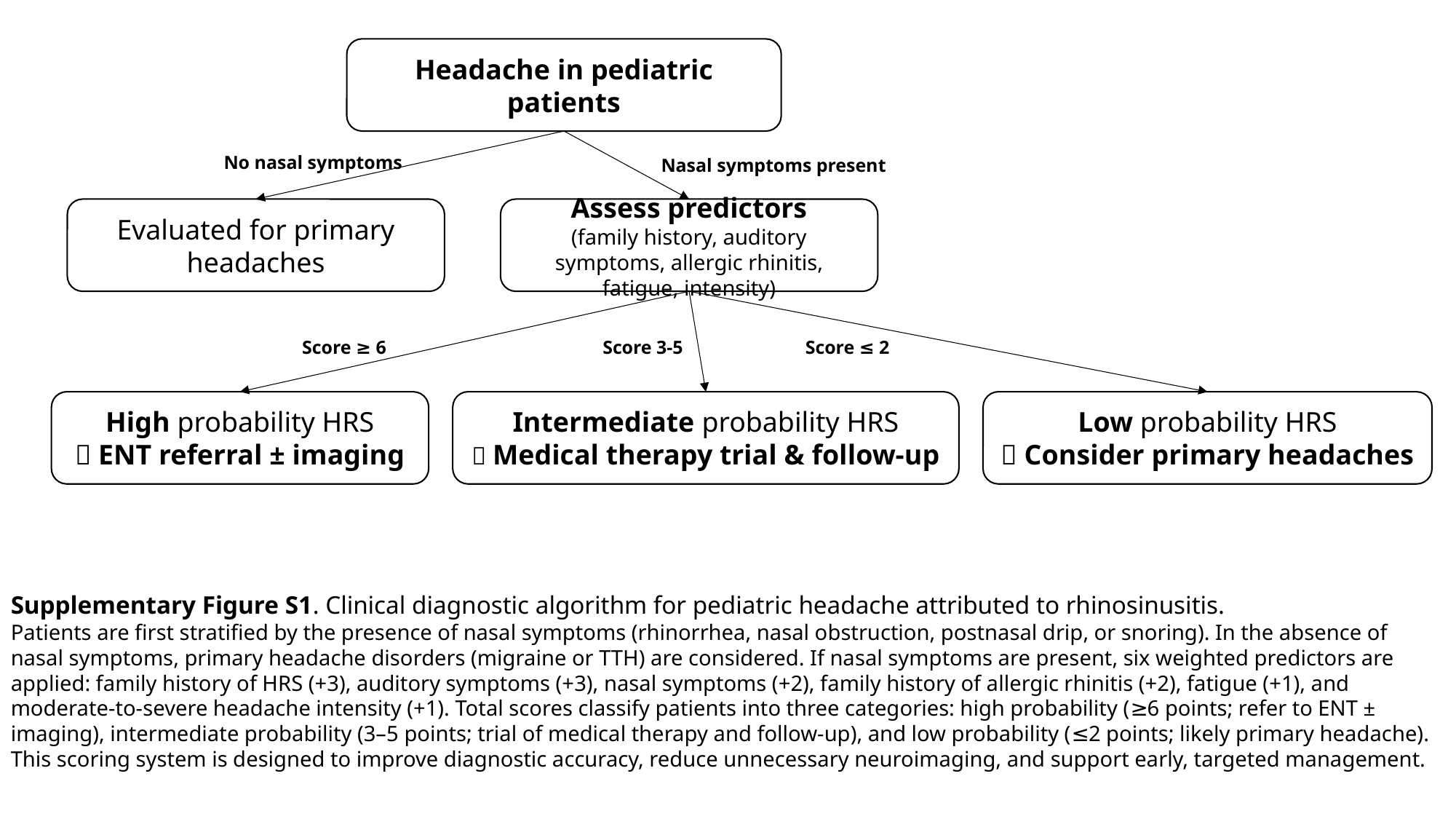

Headache in pediatric patients
No nasal symptoms
Nasal symptoms present
Assess predictors
(family history, auditory symptoms, allergic rhinitis, fatigue, intensity)
Evaluated for primary headaches
Score ≥ 6
Score 3-5
Score ≤ 2
High probability HRS
 ENT referral ± imaging
Intermediate probability HRS
 Medical therapy trial & follow-up
Low probability HRS
 Consider primary headaches
Supplementary Figure S1. Clinical diagnostic algorithm for pediatric headache attributed to rhinosinusitis.
Patients are first stratified by the presence of nasal symptoms (rhinorrhea, nasal obstruction, postnasal drip, or snoring). In the absence of nasal symptoms, primary headache disorders (migraine or TTH) are considered. If nasal symptoms are present, six weighted predictors are applied: family history of HRS (+3), auditory symptoms (+3), nasal symptoms (+2), family history of allergic rhinitis (+2), fatigue (+1), and moderate-to-severe headache intensity (+1). Total scores classify patients into three categories: high probability (≥6 points; refer to ENT ± imaging), intermediate probability (3–5 points; trial of medical therapy and follow-up), and low probability (≤2 points; likely primary headache). This scoring system is designed to improve diagnostic accuracy, reduce unnecessary neuroimaging, and support early, targeted management.
