# Supplementary material for: Clinical and Multivariate Predictors of Headaches Attributed to Rhinosinusitis in Pediatric Patients: A Comparative Study with Migraine and Tension-Type Headache
Source: Children (Basel). 2025 Nov 17;12(11):1557. doi: 10.3390/children12111557 (PMC12651926; doi:10.3390/children12111557)
Supplement: Supplementary file 1 [file children-12-01557-s001.zip › Supplement Table S1_Children.pdf]

Supplement Table S1. Comparison of headache characteristics between the migraine, TTH and HRS.

Characteristics	Migraine (n = 1140)	TTH (n = 474)	HRS (n= 137)	*p* value
Male (n, %)	471 (41.3)	232 (48.9)	118 (58.1%)	<0.001
Female (n, %)	669 (58.7)	242 (51.1)	85 (41.9%)	
Age (years), median (interquartile range)	12 (2-18)	11 (3-18)	9 (3-17)	<0.001
Age at diagnosis (n, %)				<0.001
Pre-school age (≤6 years)	81 (7.1)	62 (13.1)	39 (28.5)	<0.001
Children (7–12 years)	450 (39.5)	232 (48.9)	78 (56.9)	<0.001
Adolescent (13–18 years)	609 (53.4)	180 (38.0)	20 (14.6)	<0.001
Onset type (n, %)				<0.001
Acute (≤3 months)	221 (19.4)	65 (13.7)	43 (31.4)	<0.001
Acute recurrent (≤3 months)	227 (19.9)	189 (39.9)	19 (13.9)	<0.001
Chronic non-progressive (>3 months)	214 (18.8)	148 (31.2)	50 (36.5)	<0.001
Chronic progressive (>3 months)	478 (41.9)	72 (15.2)	25 (18.2)	<0.001
Localization (n, %)				<0.001
Diffuse	144 (12.6)	107 (22.6)	20 (14.6)	<0.001
Localized	956 (83.9)	357 (75.3)	97 (70.8)	<0.001
Mixed	40 (3.5)	10 (2.1)	20 (14.6)	<0.001
Duration (n, %)				<0.001
<30 minutes	146 (12.8)	132 (27.9)	38 (27.7)	<0.001
30–<60 minutes	141 (12.4)	93 (19.6)	35 (25.6)	<0.001
≥1 hour	853 (74.8)	249 (52.5)	64 (46.7)	<0.001
Frequency (n, %)				<0.001
<2/month	131 (11.5)	60 (12.7)	0	<0.001
2–<4/month	248 (21.8)	96 (20.3)	49 (35.8)	<0.001
4–<15/month	298 (26.2)	146 (30.8)	50 (36.5)	0.319
≥15/month	161 (14.1)	67 (14.1)	38 (27.7)	<0.001
Daily	151 (13.2)	105 (22.1)	0	<0.001
Intensity (n, %)				<0.001
Mild	80 (7.0)	175 (36.9)	18 (13.1)	<0.001
Moderate	749 (65.7)	261 (55.1)	83 (60.6)	<0.001
Severe	311 (27.3)	38 (8.0)	36 (26.3)	<0.001
Sleep disturbance due to headache (n, %)	158 (13.9)	53 (11.2)	22 (16.1)	0.217
Morning headache (n, %)	368 (32.3)	113 (23.8)	33 (24.1)	0.001
Characteristics (n, %)				<0.001
Throbbing	727 (63.8)	21 (4.4)	34 (24.8)	<0.001
Sharp	80 (7.0)	1 (0.2)	9 (6.6)	<0.001
Cramping	24 (2.1)	3 (0.6)	1 (0.7)	0.069
Prickling	105 (9.2)	27 (5.7)	10 (7.3)	0.058
Constant/dull	33 (2.9)	83 (17.5)	10 (7.3)	<0.001
Pressure	192 (16.8)	345 (72.8)	33 (24.1)	<0.001
Mixed	34 (3.0)	7 (1.5)	39 (28.5)	<0.001
Others	49 (4.3)	8 (1.7)	1 (0.7)	0.006
Accompanied symptoms (n, %)	998 (87.5)	168 (35.4)	78 (56.9)	<0.001
Nausea/vomiting	796 (69.8)	22 (4.6)	69 (50.4)	<0.001
Abdominal pain	66 (5.8)	15 (3.2)	15 (11.0)	<0.001
Photophobia	266 (23.3)	29 (6.1)	12 (8.8)	<0.001

Phonophobia	279 (24.5)	43 (9.1)	0	<0.001
Dizziness	394 (34.6)	96 (20.3)	44 (32.1)	<0.001
Nasal symptoms*	21 (1.8)	11 (2.3)	53 (38.7)	<0.001
Neurologic manifestations (n, %)	286 (25.1)	37 (7.8)	30 (21.9)	<0.001
Gait disturbance	1 (0.09)	0	2 (1.5)	0.223
Focal weakness	33 (2.9)	4 (0.8)	3 (2.2)	<0.001
Visual disturbance	224 (19.6)	21 (4.4)	26 (19)	<0.001
Hearing impairment	10 (0.9)	1 (0.2)	10 (7.3)	0.003
Dysarthria/aphasia	9 (0.8)	0	1 (0.7)	<0.001
Dysesthesia	38 (3.3)	2 (0.4)	2 (2.5)	<0.001
Decreased consciousness	10 (0.9)	5 (1.1)	2 (2.5)	0.013
Seizure	3 (0.3)	1 (0.2)	0	0.072
Movement symptom†	17 (1.5)	3 (0.6)	4 (2.9)	<0.001
Triggering factors (n, %)	266 (23.3)	109 (23)	25 (18.2)	0.005
Emotional stress	182 (16.0)	79 (16.6)	15 (11.0)	0.256
Hunger	7 (0.6)	0	0	0.152
Weather	37 (3.2)	15 (3.2)	2 (2.5)	0.157
Fatigue	43 (3.8)	24 (5.1)	15 (11.0)	<0.001
Exercise	17 (1.5)	8 (1.7)	4 (2.9)	0.463
Light	11 (1.0)	0	2 (1.5)	0.071
Noise	8 (0.7)	2 (0.4)	0	0.518
Smell	16 (1.4)	8 (1.7)	0	0.322
Season at diagnosis (n, %)				0.093
Spring	288 (25.3)	107 (22.6)	35 (25.6)	1.000
Summer	365 (32.0)	133 (28.0)	45 (32.8)	1.000
Fall	296 (26.0)	125 (26.4)	30 (21.9)	1.000
Winger	191 (16.7)	109 (23.0)	27 (19.7)	1.000
Family history of migraine (n, %)	368 (32.3)	30 (6.8)	9 (6.6)	<0.001
Family history of TTH (n, %)	45 (3.9)	114 (24.1)	3 (2.2)	<0.001
Family history of HRS (n, %)	12 (1.0)	5 (1.1)	36 (26.3)	<0.001
Family history of allergic rhinitis (n, %)	23 (2.0)	6 (1.3)	20 (14.6)	<0.001

†Tremor, myoclonus; *Rhinorrhea, nasal stuffiness, postnasal drip, snoring. TTH: tension-type headache, HRS: headache attributed to acute rhinosinusitis
