# Supplementary material for: Clinical and Multivariate Predictors of Headaches Attributed to Rhinosinusitis in Pediatric Patients: A Comparative Study with Migraine and Tension-Type Headache
Source: Children (Basel). 2025 Nov 17;12(11):1557. doi: 10.3390/children12111557 (PMC12651926; doi:10.3390/children12111557)
Supplement: Supplementary file 1 [file children-12-01557-s001.zip › Supplement table S2_Children.pdf]

Supplement Table S2. Clinical differences between migraine, TTH, and HRS

Clinical features	Migraine	TTH	HRS
Typical age group	School-aged to adolescent	School-aged to adolescent	Preschool to school-aged
Sex predominance	Slight female	Balanced	Male predominance
Headache duration	>1hour, often prolonged	30 minutes - several hours	Usually < 1hour
Intensity	Moderate to severe	Mild to moderate	Moderate to severe
Location	Frontal or temporal, often unilateral	Bilateral or diffuse	Frontal or maxillary, often pressure-like
Quality of pain	Throbbing, pulsating	Pressing, tight band-like	Pressure, dull, occasionally throbbing
Associated symptoms	Nausea, photophobia, phonophobia	Rare systemic symptoms	Nasal congestions, rhinorrhea, postnasal drip, auditory symptoms
Triggers	Stress, fatigue, fasting, light/noise	Emotional stress, poor posture	Upper respiratory infection, allergy
Family history	Migraine common	Less common	Allergic rhinitis, HRS common
Response to treatment	Triptans, NSAIDs	NSAIDs	Nasal steroids, antibiotics (if bacterial)
Diagnostic clue	Sensory hypersensitivity	Muscle tension	Nasal or auditory involvement, short duration

TTH: tension-type headache, HRS: headache attributed to acute rhinosinusitis
